# Plant-Associated Bacterial Degradation of Toxic Organic Compounds in Soil

**DOI:** 10.3390/ijerph6082226

**Published:** 2009-08-12

**Authors:** Martina McGuinness, David Dowling

**Affiliations:** Department of Science and Health, Institute of Technology Carlow, Kilkenny Road, Carlow, Ireland; E-Mail: Martina.McGuinness@itcarlow.ie

**Keywords:** toxic organics, soil, bacteria, bioremediation

## Abstract

A number of toxic synthetic organic compounds can contaminate environmental soil through either local (e.g., industrial) or diffuse (e.g., agricultural) contamination. Increased levels of these toxic organic compounds in the environment have been associated with human health risks including cancer. Plant-associated bacteria, such as endophytic bacteria (non-pathogenic bacteria that occur naturally in plants) and rhizospheric bacteria (bacteria that live on and near the roots of plants), have been shown to contribute to biodegradation of toxic organic compounds in contaminated soil and could have potential for improving phytoremediation. Endophytic and rhizospheric bacterial degradation of toxic organic compounds (either naturally occurring or genetically enhanced) in contaminated soil in the environment could have positive implications for human health worldwide and is the subject of this review.

## 1. Introduction

Synthetic organic compounds are ubiquitous in our modern environment. They are found in our homes, workplaces, public spaces and in agriculture. These organic compounds can enter soil, air and water through either local or diffuse contamination and can often be found far from their source of origin. Local or point source pollution involves discrete locations of pollution, e.g., industrial waste via factory or sewer pipes. Diffuse or nonpoint source pollution involves pollution from multiple cumulative inputs over a large area, e.g., agricultural waste (from farms) and municipal waste. While a large number of synthetic organic compounds are harmless, some are toxic and pose serious environmental and human health risks. Effects of contamination of environmental soil with toxic synthetic organic compounds include the poisoning of animals and plants, altering of ecosystems, and human health risks. International and national legislation attempts to address local sources of pollution by targeting industrial discharge. However, diffuse sources of pollution are more difficult to monitor and usually have a greater impact on the quality of the environment.

Many toxic synthetic organic compounds are persistent and are stored in fat tissue, due to their hydrophobic properties, resulting in bioaccumulation. Therefore, organisms at higher levels in food chains (e.g., humans) tend to have greater concentrations of these bioaccumulated toxins stored in their fat tissue than those at lower levels resulting in biomagnification of the physiological effects of the toxins in higher organisms. At the highest level in the food chain, i.e., humans, these toxic organic compounds can be passed from mother to child either *in utero* via the placenta or *post-natally* via breast milk.

Synthetic organic compounds of concern as environmental contaminants include polychlorinated biphenyls (PCBs), pesticides, industrial solvents, petroleum products, dioxins and furans, explosives, and brominated flame retardants. Twelve organic compounds were listed as persistent organic pollutants (POPS) by the Stockholm Convention on Persistent Organic Pollutants, under the auspices of the United Nations Environment Programme (UNEP), an international agreement enforced in 2004 [1]. The 12 POPs listed by the Stockholm Convention include PCBs, nine chlorinated organic pesticides [aldrin, chlordane, dichlorodiphenyltrichloroethane (DDT), dieldrin, endrin, mirex, heptachlor, hexachlorobenzene, and toxaphene], and dioxins and furans. Although the use of these POPs worldwide has been generally phased out because of their toxicity and persistence, they can still be found as contaminants in the natural environment due to their past use and continue to pose a threat to human health.

Traditional technologies routinely used for the remediation of contaminated environmental soil include excavation, transport to specialized landfills, incineration, stabilization and vitrification. Recently, however, there has been much interest in bioremediation technologies which use plants and microorganisms (including bacteria) to degrade toxic contaminants in environmental soil into less-toxic and/or non-toxic substances. The United States Environmental Protection Agency (USEPA) defines bioremediation as a treatability technology which uses biological activity to reduce the concentration and/or toxicity of a pollutant. Bioremediation technologies offer many advantages over traditional remediation technologies as they can be applied *in situ* without the need for removal and transport of contaminated soil, are usually less expensive and less labour-intensive relying on solar energy, have a lower carbon footprint, and have a high level of public acceptance. Phytoremediation, the use of plants to degrade toxic contaminants in the environment involving a number of processes including phytoextraction, phytotransformation, phytostabilization, phytovolatilization and rhizofiltration, has been reviewed extensively and the reader is directed to a number of recent reviews [2–4]. Phytoextraction (or phytoaccumulation) involves the uptake and concentration of pollutants into harvestable biomass for sequestration or incineration. Phytotransformation involves enzymatic modification resulting in inactivation, degradation (phytodegradation), or immobilization (phytostabilization) of pollutants. Phytovolatilization involves the removal of pollutants from soil and their release through leaves via evapotranspiration processes and rhizofiltration involves the filtering of water through a mass of roots to remove pollutants. While some success has been reported using plants alone in bioremediation [2–4], the use of plants in conjunction with plant-associated bacteria offers much potential for bioremediation. Degradation of toxic organic compounds in environmental soil by plant-associated bacteria can involve endophytic and rhizospheric bacteria. Endophytic bacteria are non-pathogenic bacteria that occur naturally in the internal tissues of plants and can promote plant growth, be beneficial to the plant host by producing a range of natural products, and contribute to enhanced biodegradation of environmental soil pollutants [5,6]. Almost all 300,000 plant species identified have at least one species of endophyte [7]. Endophytic bacterial species isolated from plants, to date, include *Acetobacter, Arthrobacter, Bacillus, Burkholderia, Enterobacter, Herbaspirillum and Pseudomonas,* as reviewed by Lodewyckx *et al*. [8]. In contrast to endophytes, rhizospheric bacteria are associated with the rhizosphere of plants, i.e., the area of soil surrounding plant roots, where complex microbial communities are supported by root exudates, mucilage, and decaying root cells [9]. Rhizospheric soil typically has 10–100 times more microbes, on a per gram basis, than unvegetated soil [10]. Rhizospheric microbial communities can benefit the plant by synthesizing compounds that protect plants by decreasing plant stress hormone levels, delivering key plant nutrients, protecting against plant pathogens, and degrading contaminants before they negatively impact the plants as reviewed by Gerhardt *et al*. [4]. Phytoremediation has been reported to be approximately 10-fold less expensive than traditional remediation technologies [11] and can include the use of buffers, vegetation filters, *in situ* phytoremediation plantings, and percolation controlling vegetative caps as described by Licht and Isebrands [12]. Therefore, the use of endophytic and rhizospheric bacteria capable of degrading toxic synthetic organic compounds in combination with specific plants (chosen to suit the environment to be remediated and/or because of their relationship with the endophytic or rhizospheric bacteria to be used) could offer an efficient, economic and sustainable remediation technology for the twenty first century.

The spectrum of toxic synthetic organic compounds identified as contaminants in environmental soil and the use of plant-associated endophytic bacteria and rhizospheric bacteria to degrade these toxic organic compounds will be the subject of this review. The use of biotechnology to engineer plant-associated bacteria to produce specific enzymes capable of degrading these toxic substances and the potential of these bacteria to contribute to bioremediation will also be discussed.

## 2. Toxic Synthetic Organic Compounds in Environmental Soil and Associated Human Health Risks

Because of their chemical structure many synthetic organic compounds are extremely resistant to natural breakdown processes and once released into the environment may persist for years and even decades. Many of these organic compounds are toxic and are associated with serious human health risks as described below.

### 2.1. PCBs and Synthetic Organic Pesticides

PCBs are toxic synthetic aromatic compounds notorious for their persistence and potential toxicity and were widely used in industry in the twentieth century. They are a group of polychlorinated biphenyl compounds with 209 different congeners or related chemicals, some containing up to 10 chlorine atoms. However, commercially available PCB mixtures (e.g., Arochlor) typically contain only 20–60 congeners. Because of the chemical stability, electronic insulating properties, thermal stability and non-flammability, PCBs were widely used in flame retardants, dielectric fluids in capacitors, transformers, hydraulic fluids, surface coating, adhesives and dyes. The manufacture of PCBs was banned in the USA in 1978 because of their toxicity. Although the manufacture of PCBs has been banned they remain a problem in the environment because of their persistence. Since PCBs were first synthesized in 1864, it is estimated that approximately 1 million tonnes have been manufactured worldwide [13] and that approximately 30% of all PCBs manufactured has been released into the natural environment resulting in the contamination of soils and sediments [14]. PCBs have been detected in polar bears in the Arctic, an environment far removed from industry, providing evidence of the dispersal of these toxic synthetic organic compounds in the natural environment [15,16].

DDT is one of the best known toxic chlorinated organic pesticides and although its use as an agricultural insecticide worldwide was banned by the Stockholm Convention, it is still used to control malaria in some parts of the world and remains controversial. DDT was used worldwide as an insecticide from the 1940s until the 1970s, when it was banned in the USA and other countries. In 1962, Rachel Carson in her popular book ‘Silent Spring’ suggested that DTT and other pesticides were associated with cancer and that their agricultural use was a threat to wildlife, particularly birds [17]. Along with the passing of the Endangered Species Act in 1973, the US ban on DTT is cited by scientists as a major factor in the comeback of the bald eagle in the US [18]. However, when a global ban on DDT was proposed in 2001, several countries in Africa claimed that DDT was still needed as an inexpensive and effective means for control of the vector associated with malaria. Although DDT is generally not toxic to human beings and was banned mainly for ecological reasons, subsequent research has shown that exposure to DDT at amounts that would be needed to control malaria might cause preterm birth and early weaning, eliminating the benefit of reducing infant mortality from malaria [19,20]. Therefore, the use of DDT to help control malaria remains controversial because of its associated human health risks [19,20].

Two of the most commonly used pesticides in agriculture worldwide, the chlorinated organic compounds 2,4-dichlorophenoxyacetic acid (2,4-D) and atrazine, are not listed by the Stockholm Convention as POPs but have been listed by the USEPA as toxic and are associated with human health risks [21]. Both 2,4-D and atrazine are broad-leaf herbicides protecting many of the world’s important crops, such as wheat, corn, and rice, which are cereal grains. Since its introduction in 1946, 2,4-D remains the most widely used herbicide worldwide. Although 2,4-D is biodegradable, it may persist in soil and water if microbes with the required capacity for biodegradation are not present in sufficient numbers. Atrazine has been banned by the European Union since 2007, but is still used in many parts of the world, and can remain in soil for greater than one year after use and leach into groundwater contaminating private and community wells [22].

Other synthetic chlorinated organic pesticides of concern as contaminants of environmental soil include tetrachlorophenol (TCP), pentachlorophenol (PCP), and the tin-containing pesticide, tributylin (TBT). TCP is an insecticide and a bactericide and is widely used as a preservative for latex, wood, and leather. PCP is a disinfectant, a fungicide, and an extremely effective preservative for wood. In addition, PCP and its products are toxic to plants, facilitating their use as defoliants and general herbicides. TCP and PCP can be released into the environment as a result of their manufacture, storage, transport, or use as an industrial wood preservative. Their use at sawmills has lead to extensive groundwater contamination [23]. TCP and PCP are strong irritants and can produce skin and eye irritation upon contact. They are readily absorbed through the skin and can produce systemic effects. Acute exposure to TCP and PCP in animals is associated with convulsant activity and inhibition of oxidative phosphorylation [24]. The tin-containing biocide TBT is used to control a wide variety of organisms. It is used in wood preservatives, as an anti-fouling pesticide in marine paints, and as an antifungal agent in industrial water systems. TBT compounds bioaccumulate as they move up the marine food chain and have been associated with toxicity to a number of marine organisms such as molluscs, otters, dolphins and whales [25,26]. Chlorobenzoates, toxic metabolic intermediates produced from biodegradation of a variety of compounds including PCBs and chlorinated aromatic pesticides, are also considered environmental contaminants.

Glyphosate is an organophosphate broad-spectrum herbicide originally sold in the 1970s under the tradename Roundup^™^. Today, glyphosate is the most widely used herbicide in the USA. Although glyphosate is less toxic than chlorinated organic pesticides, it is a suspected endocrine disruptor. A review of at least 58 studies on the effects of Roundup^™^ suggests that non-target organisms were exposed to only minimal acute and chronic risk [27]. However, more recent research reports that glyphosate induces a variety of functional abnormalities in the specific activity of the enzymes in the liver, heart and brain, in pregnant rats and their fetuses [28]. Glyphosate was also reported to interfere with an enzyme involved in testosterone production in mouse cell culture [29] and to interfere with an oestrogen biosynthesis enzyme in cultures of human placental cells [30].

A number of synthetic organic compounds, e.g., PCBs and many pesticides, are suspected endocrine disruptors and have been associated with the feminization of males. A number of scientists attribute the striking drop in sperm counts among men worldwide to these endocrine disruptors. In a landmark study, Danish researchers reviewed 61 studies and reported in 1992 that the number and motility of sperm in men’s semen had declined by 50% since 1938 [31]. Other studies have linked endocrine disruptors and rising rates of testicular cancer [32] and breast cancer [33,34]. Exposure of humans to PCBs and a number of chlorinated organic pesticides has been associated with an increased risk of developing cancer [35] and with developmental disabilities [36] in children. A recent study reported an association between exposure to pesticides and an increased incidence of Parkinson’s disease [37].

### 2.2. Volatile Organic Compounds (VOCs)

VOCs are vapours emitted by various solids or liquids, e.g., petrol, diesel, pesticides, paint, cleaning supplies and adhesives, many of which have short- and long-term adverse health effects. Benzene, toluene, ethylbenzene and xylene (BTEX) compounds are a family of VOCs based on the benzene structure and are found in petroleum products. Refineries adjust the amounts of BTEX compounds in petrol so as to meet vapour pressure and octane standards. BTEX compounds are major contaminants of environmental soil and groundwater and are usually found near petroleum and natural gas production sites, petrol stations and other sites with underground or above-ground storage tanks containing petroleum products. Exposure of humans to BTEX can occur by either ingestion (drinking water from contaminated wells), or by inhalation (exposure to BTEX contaminated water via showering or laundering). Acute exposure to petrol and its BTEX components has been associated with skin and sensory irritation, central nervous system depression, and effects on the respiratory system in humans while long-term exposure to BTEX compounds affects the kidney, liver and blood systems [38]. According to the USEPA, there is evidence from both human epidemiological and animal studies that benzene is a human carcinogen, and that workers exposed to high levels of benzene in occupational settings were found to have increases rates of leukaemia [39].

Methyl tertiary butyl ether (MTBE), also a VOC, is used as a fuel oxygenate, i.e., a chemical containing oxygen that is added to fuels, especially petrol, to make them burn more efficiently. It can be a major contaminant of groundwater as a result of the widespread spillage or leakage of MTBE-containing petrol from underground storage tanks at petrol stations. The USEPA concluded that MTBE was a potential human carcinogen at high doses [40].

Trichloroethylene (TCE), and tetrachloroethene [also known as perchloroethylene (PCE)], are chlorinated VOCs. TCE was widely used as a volatile anaesthetic and also as an industrial solvent during the first half of the twentieth century. As an anaesthetic, TCE was originally thought to be less toxic to the liver than chloroform, and to be less pungent and flammable than ether. However, TCE was subsequently found to be associated with serious health risks and was replaced as an anaesthetic by halothane in the 1950s. The symptoms of acute exposure to TCE are similar to those of alcohol intoxication, beginning with headache, dizziness, and confusion, progressing with increasing exposure to unconsciousness [41]. Much of what is known about the human health effects of TCE is based on occupational exposures. Beyond the effects to the central nervous system, workplace exposure to TCE has been associated with toxic effects in the liver and kidney [41]. Over time, occupational exposure limits on TCE have tightened, resulting in more stringent ventilation controls and personal protective equipment use by workers. TCE was also used as a dry cleaning solvent until it was replaced by PCE in the 1950s. More recently, TCE was used as a cleaning solvent to clean military weapons during the Gulf War. As a result of this exposure of military personnel to TCE, an association with the neurological disorder amyotrophic lateral sclerosis (Lou Gehrig’s disease) was reported by Kasarskis *et al*. [42], and an association with a neurologic syndrome resembling Parkinson’s disease by Gash *et al*. [43]. PCE is still widely used as a solvent in the dry-cleaning industry and is a common environmental soil contaminant, associated with central nervous system dysfunction [44]. PCE contamination of soil usually results from spillage, overfilling, sewer leakage, or illegal disposal by commercial dry cleaning facilities. Because of the mobility of PCE in groundwater, its toxicity at low levels, and its density (which causes it to sink below the water table), cleanup activities tend to be especially problematic compared to the cleanup of oil spills.

### 2.3. Hydrocarbons

Hydrocarbons contain hydrogen and carbon, and can be found in the environment as gases, tiny particles, or droplets. Hydrocarbons, primarily measured as total petroleum hydrocarbons, are the majority of organic compounds in most crude oils and contain hundreds of individual components. Most hydrocarbons in the environment are associated with the use of petrol, diesel, crude oil, and oil products in vehicles used for transportation. Hydrocarbons can be gases (e.g., methane and propane), liquids (e.g., hexane and benzene), waxes or low melting solids (e.g., paraffin wax and naphthalene), or polymers (e.g., polyethylene, polypropylene and polystyrene). There are three major categories of aromatic hydrocarbons of concern as contaminants of environmental soil. They are: (i) polycyclic aromatic hydrocarbons (PAHs), (ii) heterocyclic aromatic hydrocarbons, and (iii) alkyl PAHs, as described in more detail below.

#### 2.3.1. Polycyclic Aromatic Hydrocarbons (PAHs)

All PAHs contain at least two fused aromatic rings in linear, angular or cluster arrangements [45,46] and can be produced by petroleum production sites and combustion processes. PAHs are generally more difficult to degrade than many other organic compounds and are persistent in environmental soil. The USEPA has listed 16 PAHs as priority pollutants because of their persistence and carcinogenicity based on toxicity, potential for human exposure, frequency of occurrence at hazardous waste sites, and the extent of information available [47]. The USEPA considers seven of these 16 priority PAHs as probable human carcinogens [48]. They are benzo(a)anthracene, chrysene, benzo(a)pyrene, benzo(b)fluoranthene, benzo(k)fluoranthene, dibenz(a,h)anthracene, and indeno(1,2,3-cd)pyrene. Naphthalene, a PAH with two fused benzene rings, is produced commercially from either coal tar or petroleum. It is used mainly in the production of other chemicals including plasticizers, dyes, and insecticides, and is a major component of creosote and mothballs. Exposure to naphthalene is associated with haemolytic anaemia (abnormal breakdown of red blood cells) [49], cataracts and retinal damage [50].

#### 2.3.2. Heterocyclic Aromatic Hydrocarbons: Dioxins and Furans

Heterocyclic aromatic hydrocarbons include dioxins and furans, both listed by the Stockholm Convention as POPs [1]. Dioxins are produced unintentionally by industry due to incomplete combustion, as well as during the manufacture of certain pesticides and other chemicals, metal recycling and pulp and paper bleaching. Dioxins have also been found in automobile exhaust, tobacco smoke and wood and coal smoke and in commercial mixtures of PCBs. Dioxins are a group of 75 related chemical compounds known as polychlorinated dibenzo-*p*-dioxins. Each of the 75 compounds differs in the number and location of chlorine atoms on a basic three-ringed structure of carbon, hydrogen and oxygen atoms. Furans are a group of 135 related heterocyclic aromatic hydrocarbons called polychlorinated dibenzofurans. Of these, 17 [including the most toxic, 2,3,7,8-tetrachloro-dibenzo-p-dioxin (2,3,7,8-TCDD)] pose a major health risk. Similar in chemical structure and biological properties, dioxins and furans are usually found together in the environment as complex mixtures. The toxicity of each compound depends on the number and position of the chlorine atoms within the molecules. 2,3,7,8-TCDD was the contaminant in the weedkiller 2,4,5-trichlorophenoxy-acetic acid (2,4,5-T) used by the U.S. military as a defoliant in the early 1970s in the Vietnam War in ‘Agent Orange’. Agent Orange was equal parts 2,4,5-T and 2,4-D (see section 2.1). Dioxins and furans do not dissolve in water and can attach to particles of soil, dust and sediment. As a result, they can persist unchanged in the environment, mainly in soil and sediment, for years. A number of studies have been carried out on populations after accidental environmental exposure to high levels of dioxins and furans and report that chloracne, a skin disorder, is the most common human health effect [51]. The facial images of the Ukranian presidential candidate, Victor Yuschenko, widely circulated by the media in 2004 clearly showed the effect of chloracne as a result of deliberate 2,3,7,8-TCDD poisoning. Extreme exposures also lead to other effects on the skin, liver, immune system, reproduction system, and the central nervous system [52].

#### 2.3.3. Alkyl PAHs

Alkyl PAHs are PAHs with alkyl group substitution on their ring structures. The alkyl groups generally have one to four saturated carbon atoms, and thus can produce many different structural isomers. Alkylated PAHs are more abundant, persist for a longer time, tend to bioaccumulate to a greater degree (alkyl substitution usually decreases water solubility), and are sometimes more toxic than the parent PAHs. Within an aromatic series, acute toxicity increases with increasing alkyl substitution on the aromatic nucleus. Crude oils contain primarily the alkyl homologues of aromatic compounds and relatively small quantities of unsubstituted PAHs. Usually, the most significant compounds when assessing environmental damage associated with oil spills are PAHs and alkylated PAHs.

### 2.4. Explosives

Organic explosives including trinitrotoluene (TNT), hexahydrotrinitrotriazine or Royal Demolition Explosive (RDX), and octahydro-tetranitrotetraocine or High Melting eXplosive, (HMX) can contaminate environmental soil. TNT has been associated with aplastic anaemia and hepatitis, while RDX has been shown to affect the central nervous system [53]. While information on the health risks associated with HMX is limited, studies in laboratory rats, mice, and rabbits indicate that HMX may be harmful to the liver and central nervous system [54].

### 2.5. Brominated Flame Retardants

Brominated flame retardants are POPs and are the most widely used flame retardants because of their efficiency and low production costs [55]. The major brominated flame retardants used worldwide are tetrabromobisphenol A, hexabromocyclododecane, and polybromodiphenyl ethers [55]. These compounds can enter the environment locally via wastewaters of industrial facilities, through volatilization, leaching and combustion. Flame retardants have been found in air, water, soils and sediments far from where they are produced or used [55–57], again providing evidence of the wide dispersal of toxic synthetic organic compounds in the environment.

Reducing the levels of toxic synthetic organic compounds in the environment is an issue of growing concern as the effects of these chemicals on human heath become more widely understood. Therefore, the use of plant-associated endophytic and rhizospheric bacteria to degrade toxic organic compounds in contaminated environmental soil could have positive implications for human health worldwide.

## 3. Remediation Technologies

### 3.1. Traditional Technologies for the Remediation of Contaminated Soil

It is estimated that traditional global remediation costs are in the range of $US25-50 billion annually [58,59]. Unfortunately, this high cost of remediation contributes to the abandonment worldwide of a large number of polluted commercial sites or brownfields. For example, in the USA, the USEPA Office of Underground Storage Tanks reported that 34% of known contaminated sites choose to be in non-compliance in 2008 [60]. Some of the reasons for non-compliance are typically (i) non-compliance has a lower immediate cash cost than compliance using traditional remediation technologies, and (ii) the demand for green remediation is not yet powerful enough to drive action and does not impact on sales and revenue. However, with: (i) an increasing public awareness of the need to move towards a low carbon economy, (ii) the introduction of regulations with an increased emphasis on a low carbon economy, and (iii) the development of sustainable bioremediation metrics, there is an increased interest in moving away from traditional technologies for the remediation of contaminated soil (e.g., excavation, transport to specialized landfills, incineration, stabilization and vitrification) towards bioremediation technologies by regulators, consultants and representatives from industry.

### 3.2. Bioremediation Technologies

In remediation of the environment, bioremediation is a treatment process that uses microorganisms (including bacteria) and plants to degrade toxic contaminants into less toxic or non-toxic substances. According to the U.S. Sustainable Remediation Forum (US SURF), sustainable remediation is broadly defined as a remedy or combination of remedies whose net benefit on human health and the environment is maximized through the judicious use of limited resources [61]. Because bioremediation technologies can be applied *in situ* without the need for removal and transport of contaminated soil, are usually less expensive and less labour-intensive (relying on solar energy), have a lower carbon footprint, and have a high level of public acceptance than traditional remediation technologies, they potentially offer a sustainable solution to the problem of contaminated environmental soil. However, conditions in the contaminated environmental soil need to be optimized for effective biodegradation of the target contaminants, i.e., the levels of moisture, pH and temperature in the soil will dictate survival ranges for microorganisms and plants used for bioremediation, abundant oxygen will facilitate mineralization of soil contaminants, concentrations of nutrient and hydrocarbons in the soil will need to be balanced for efficient bioremediation, and suitable microorganisms and plants will be required to degrade and/or mineralize target contaminants. Although it appears that the advantages associated with the use of bioremediation technologies clearly outweigh the disadvantages, when compared to traditional remediation technologies, other factors to consider when using bioremediation technologies include the length of time required (months or years), geographic limitations on the use of specific plants, and the seasonal limitations associated with the use of specific plants. Choosing a technology for sustainable remediation of contaminated environmental soil requires detailed analyses of the environmental impact. Sustainable remediation metrics include economic, societal and environmental metrics for comparing and selecting remedies and monitoring success and include important elements such as water use, worker safety, community impact, and the net environmental benefit [61]. Bioremediation technologies compare favourably with traditional remediation technologies when analysed using sustainability remediation metrics

## 4. Biodegradation of Toxic Organic Compounds in Environmental Soil

A number of bacterial strains have been identified in a wide variety of contaminated environments with enzymes capable of degrading toxic organic compounds. Anaerobic bacteria can convert highly chlorinated PCB congeners into less chlorinated biphenyls by reductive dechlorination [62,63]. Aerobic bacteria, e.g., *Burkholderia xenovorans* LB400 [64] and *Rhodococcus* sp. strain RHA1 [65], can then cleave lesser chlorinated biphenyl rings to yield chlorinated benzoates and pentanoic acid derivatives which are often degradable by other bacteria. The dechlorinating bacteria *Dehalococcoides ethenogenes, Dehalobacter restrictus, Desulfitobacterium dehalogenans, Dehalospirillum multivorans, Desulfuromonas chloroethenica,* and *Desulfomonile tiedjei* are capable of dehalogenating PCE [66] and other chlorinated aromatic compounds [67]. *Dehalococcoides ethenogenes* strain 195, is the only bacterial strain which completely dechlorinates PCE to yield ethylene and is of interest because of its potential use in the bioremediation of TCE and PCE contaminated sites [68]. Mannisto *et al.* [69] identified bacterial strains *Herbaspirullum* sp K1, *Sphingomonas* strains K74 and MT1, *Nocardioides* sp K44, that could degrade TCP faster at low temperature than at room temperature. *Sphingobium chlorophenolicum* strain ATCC 39723 can completely mineralize PCP [70]. Bacteria involved in the biodegradation of petroleum products in a number of different environmental soil types have also been identified [71,72]. However, for the purpose of this review article only plant-associated endophytic and rhizospheric bacteria associated with the degradation of toxic organic compounds in the environment will be discussed.

### 4.1. Endophytic Bacteria and Phytoremediation

A number of endophytic bacteria reported to contribute to degradation of environmental pollutants *in planta* are listed in Table 1.

Plant-associated endophytes with potential for bioremediation identified, to date, include endophytes of poplar trees as shown in Table 1. Van Aken *et al*. [81,82] describe a methylotrophic endophytic bacterium isolated from hybrid poplar trees (*Populus deltoides* X *Populus nigra* DN34) that was capable of degrading the explosives TNT, RDX and HMX, mineralizing approximately 60% of the RDX and HMX to carbon dioxide in approximately two months, suggesting that these endophytes may have potential for remediation of environmental soil containing these explosive nitroaromatic compounds. Endophytes isolated from hybrid poplar trees (*P. trichocarpa* X *P. deltoides* cv. Hazendens and Hoogvorst) growing on a BTEX-contaminated site in Belgium have been shown to be capable of degrading VOCs (toluene and naphthalene) as well as a chlorinated organic herbicide (2,4-D) [75,78,79]. Porteus Moore *et al.* [79] described 121 endophytic strains isolated from these hybrid poplar trees, and identified 34 of these strains as having potential to enhance phytoremediation. Germaine *et al*. [75] reported that when pea (*Pisum sativum*) plants were inoculated with *Pseudomonas* endophytes, isolated from hybrid poplars (*P. trichocarpa* X *P. deltoides* cv. Hoogvorst) and capable of degrading 2,4-D, the pea plants showed no accumulation of 2,4-D in their tissues and showed little or no signs of phytotoxicity when compared to uninoculated controls suggesting that these endophytes have potential for bioremediation of environmental soil contaminated with 2,4-D.

In a recent review, Ryan *et al*. [73] listed some of the advantages associated with the use of endophytic bacteria in phytoremediation of contaminated environmental soil when compared with the use of plants alone. They include (i) quantitative gene expression of bacterial pollutant catabolic genes can be used to assess the efficiency of the remediation process, (ii) genetic engineering of a bacterial catabolic pathway is easier to manipulate than a plant catabolic pathway, and (iii) toxic pollutants taken up by the plant may be degraded *in planta* by endophytic degraders reducing the toxic effects of contaminants in environmental soil on flora and fauna. However, some disadvantages associated with the use of bacteria in plant-associated bioremediation of contaminated environmental soil, were also given by Ryan *et al*. [73]. They include (i) this technology is limited to shallow contaminants in environmental soil, (ii) it is slower than traditional remediation technologies, (iii) the choice of plant can mean that it is only seasonally effective, (iv) it is associated with phytotoxic effects of contaminants, and (v) there is potential for the environmental contaminants or their metabolites to enter the food chain if contaminants are not completely detoxified and if the plants are consumed by local fauna. More recently, Weyens *et al*. [83] reviewed the benefits of using plant-associated endophytes in bioremediation and emphasized that although successfully applied in several laboratory-scale experiments, the large-scale field application of this technology is limited by a number of issues including (i) the levels of contaminants tolerated by plants, (ii) limited bioavailability of organic contaminants, and (iii) unacceptable levels of evaportranspiration of VOCs into the atmosphere. Despite the disadvantages associated with the use of plant-associated endophytic bacteria to degrade toxic organic compounds in environmental soil, it is clear that there is potential for these bacteria to make a significant contribution to sustainable bioremediation. Doty [84], in a recent review, claims that a major advantage of using endophytic bacteria over rhizospheric bacteria in phytoremediation is that while a rhizospheric bacterial population is difficult to control, and competition between rhizospheric bacterial strains often reduces the number of the desired strains (unless metabolism of the pollutant is selective), the use of endophytes that naturally inhabit the internal tissues of plants reduces the problem of competition between bacterial strains.

### 4.2. Rhizospheric Bacteria and Phytoremediation (Rhizoremediation)

Rhizoremediation is a specific form of phytoremediation involving plants and their associated rhizospheric microorganisms (bacteria and fungi). Rhizoremediation can either occur naturally or can be facilitated by inoculating soil with microorganisms capable of degrading environmental contaminants. To date, a number of toxic organic compounds in soil have been successfully remediated using rhizospheric bacteria as shown in Table 2. For example, Kuiper *et al*. [85,86] reported that a grass species combined with a naphthalene-degrading *Pseudomonas* species protected the grass seed from the toxic effects of naphthalene, and the growing roots propelled the naphthalene-degrading bacteria into soil that would have been too deep in the absence of roots.

### 4.3. Enhancement of Bacterial Degradation of Toxic Organic Compounds

Using biotechnology, bacterial strains can be engineered to produce specific enzymes capable of degrading toxic organic substances. Bacteria (rhizospheric and/or endophytic) can be engineered, via natural gene transfer or recombinant DNA technology, to produce specific enzymes, capable of degrading toxic organic pollutants found in the environment. Genetic engineering of endophytic and rhizospheric bacteria for use in plant-associated degradation of toxic compounds in soil is considered one of the most promising new technologies for remediation of contaminated environmental sites.

Studies using two genetically modified strains of the rhizospheric bacteria *Pseudomonas fluorescens* F113, i.e., *Pseudomonas fluorescens* F113*rifbph* (with a single chromosomal insertion of the *bph* operon) [88] and *Pseudomonas fluorescens* F113: 1180 (with a single chromosomal insertion of the *bph* operon under the control of the *Sinorhizobium meliloti* nod regulatory system) [91] reported that (i) the modified rhizospheric bacteria colonized roots as effectively as the wildtype rhizospheric bacteria, (ii) *bph* genes were expressed *in situ* in soil, and (iii) the modified rhizospheric bacteria could degrade PCBs more efficiently than the wildtype rhizospheric bacteria, indicating considerable potential for the manipulation of the rhizosphere as a useful strategy for bioremediation. *Pseudomonas fluorescens* F113: 1180 does not contain antibiotic resistance genes from the vector making this strain more suitable for *in situ* applications. Since the *bph* element in *Pseudomonas fluorescens* f113: 1180 is stable, lateral transfer of the *bph* element to a homologous recipient would not be expected to occur at detectable frequencies in the rhizosphere [101].

Dzantor [102] recently reviewed the use of biotechnology to enhance rhizospheric microbial degradation of POPs. However, because toxic organic compounds can enter the root xylem from the soil before they are degraded, and these contaminants can remain in the xylem for up to two days [103], plant-associated endophytes genetically enhanced so as to degrade toxic organic compounds appear to offer more potential than rhizospheric bacteria for reducing phytotoxicity. Endophytic bacteria can be isolated from host plants of interest (e.g., plants native to a geographical region) and genetically enhanced to contain degradation pathways or genes to degrade target contaminants before being reinoculated back into the host plant for bioremediation purposes.

Germaine *et al.* [80] reported that a genetically enhanced endophytic strain of the poplar endophyte *Pseudomonas putida* VM1441, i.e., *Pseudomonas putida* VM1441 (pNAH7), could protect inoculated pea plants from the toxic effects of naphthalene. They also showed that inoculation of plants with this strain facilitated higher (40%) naphthalene degradation rates compared with uninoculated plants in artificially contaminated soil [80]. Barac *et al*. [76] reported that a genetically enhanced endophytic strain of the soil bacterium *Burkholderia cepacia* G4 could increase inoculated yellow lupine plant tolerance to toluene, and decrease phytovolatilization of toluene from the plant into the atmosphere by 50-70% in laboratory scale experiments. In this study, the plasmid, pTOM, which encodes a pathway for the degradation of toluene, was transferred via conjugation to the natural endophyte, providing the genes for toluene degradation. Later, Taghavi *et al.* [77] extended this work to poplar trees and showed that this degradative plasmid, pTOM, could transfer naturally, via horizontal gene transfer, to a number of different endophytes *in planta*, promoting more efficient degradation of toluene in poplar plants. Horizontal gene transfer results in the natural endophyte population having the capacity to degrade environmental pollutants without the need to establish the inoculants strain long-term. Endophytes that have been engineered by horizontal gene transfer, have the distinct advantage that they may not be considered to be genetically modified microorganisms (GMMs) and could, therefore, be exempt from current international and national GM legislation thus facilitating the testing of these microorganisms in the field at an accelerated pace.

In our laboratory, bacteria expressing a specific bacterial glutathione-S-transferase (GST) isolated from *Burkholderia xenovorans* LB400, BphK^LB400^ [wildtype and mutant (Ala180Pro)], capable of dehalogenating toxic chlorinated organic pesticides were shown to protect inoculated pea plants from the effects of a chlorinated organic pesticide, chloromequat chloride [104]. Previously, it had been shown that mutating the conserved amino acid at position 180 in BphK^LB400^ from Ala to Pro resulted in an approximate 2-fold increase in GST activity towards a number of chlorinated organic substrates tested including commonly used pesticides [104,105]. These data suggest that BphK^LB400^ [wildtype and mutant (Ala180Pro)], when inserted into endophytic or rhizospheric bacteria, could have potential for bioremediation of chlorinated organic pollutants in environmental soil.

### 4.4. Transgenic Plants and Phytoremediation

An exciting alternative to the use of plant-associated bacteria to degrade toxic organic compounds in soil is the use of recombinant DNA technology to generate transgenic plants expressing bacterial enzymes resulting in improved plant tolerance and metabolism of toxic organic compounds in soil. However, as this topic is beyond the scope of the current review, the reader is directed to a number of recent reviews where the development of transgenic plants capable of detoxifying herbicides [106], organic explosives [107], TCE [108], and PCBs [109], using bacterial genes encoding enzymes involved in the detoxification of the target organic contaminant, is described in detail.

## 5. Conclusions

Much work remains to be done in carrying out field studies based on laboratory-scale experiments before commercially viable systems are available using plant-associated endophytic and rhizospheric bacteria to degrade a wide range of toxic organic compounds of concern in environmental soil. Plant-associated endophytes may offer more potential for bioremediation than plant-associated rhizospheric bacteria since: (i) the use of endophytes that are native to the host plant reduces competition between bacterial strains and may eliminate the need for reinoculation, (ii) toxic organic contaminants can remain in the plant xylem for up to two days facilitating their degradation by endophytes, and (iii) endophytes can be isolated from host plants of interest and genetically enhanced with genes encoding degradation enzymes of interest before reinoculation for bioremediation. Emphasis should be placed, when developing bioremediation systems using plant-associated bacteria, to choose wildtype bacteria, or bacteria enhanced using natural gene transfer, to avoid the complications of national and international legislation restricting and monitoring the use of GMMs. However, with a global political shift towards sustainable and green bioremediation technologies, the use of plant-associated bacteria to degrade toxic synthetic organic compounds in environmental soil may provide an efficient, economic, and sustainable green remediation technology for our twenty first century environment.

## Figures and Tables

**Table 1.** Reported cases of successful bioremediation using endophytic bacteria (adapted from Table 2 in Ryan *et al.* [73]).

**Compound**	**Plants used**	**Microbes used**	**Reference**
**PCBs, TCP**	Wheat (*Triticum* spp.)	*Herbaspirillum* sp. K1	Mannisto *et al*. [69]
**Chlorobenzoic acids**	Wild rye (*Elymus dauricus*)	*Pseudomonas aeruginosa* R75 *Pseudomonas savastanoi* CB35	Siciliano *et al*. [74]
**Pesticide** 2,4-D	Pea (*Pisum sativum*)	*Pseudomonas putida* VM1450	Germaine *et al*. [75]
**VOCs** and toluene Toluene MTBE, BTEX, TCE	Yellow lupine (*Lupinus luteus* L.) Poplar (*Populus*) Poplar (*Populus* cv. *Hazendans* and cv. *Hoogvorst*)	*Burkholderia cepacia* G4 *Burkholderia cepacia* Bu61 (pTOM-Bu61) *Pseudomonas* sp.	Barac *et al*. [76] Taghavi *et al*. [77] Germaine *et al*. [78] Porteus-Moore *et al*. [79]
**HCs** Naphthalene	Pea (*Pisum sativum*)	*Pseudomonas putida* VM1441 (pNAH7)	Germaine *et al*. [80]
**Explosives** TNT, RDX, HMX	Poplar tissues (*Populus deltoidesnigra* DN34)	*Methylobacterium populi* BJ001	Van Aken *et al*. [81,82]

**Table 2.** Reported cases of successful bioremediation using rhizospheric bacteria (adapted from Table 1 in Liu, [87]).

**Compound**	**Plants used**	**Microbes used**	**Reference**
**PCBs**	Alfalfa (*Medicago sativa*) Sugar beet (*Beta vulgaris* L.)	*Pseudomonas fluorescens*	Brazil *et al*. [88]
	Rockcress (*Arabidopsis*)	*Pseudomonas putida* Flav1-1 *Pseudomonas putida* PML2	Narasimhan *et al*. [89]
	Switchgrass (*Panicum virogatum* L.)	Indigenous degraders	Chekol *et al*. [90]
	Alfalfa (*Medicago sativa*) Sugar beet (*Beta vulgaris* L.)	*Pseudomonas fluorescens*	Villacieros *et al*. [91]
**Pesticides**			
2,4-D	Barley (*Hordeum sativum* L.)	*Burkholderia cepacia*	Jacobsen *et al*. [92]
	Red Clover (*Trifolium pratense*) Ryegrass (*Lolium perenne* L.)	Indigenous degraders	Shaw *et al*. [93]
PCP	Ryegrass (*Lolium perenne* L.)	Indigenous degraders	He *et al*. [94]
**VOCs**			
TCE	Wheat (*Triticum spp*.)	*Pseudomonas fluorescens*	Yee *et al*. [95]
**HCs**			
Petroleum products	White mustard (*Sinapsis alba* L.)	Indigenous degraders	Liste *et al*. [96]
Crude oil	Wheat (*Triticum spp*.)	*Azospirillum lipoferum* spp	Muratova *et al*. [97] Shaw *et al*. [93]
PAHs	Tall fescue grass (*Festuca arundinacea*)	*Azospirillum brasilense* Cd *Enterobacter cloacae* CAL 2 *Pseudomonas putida* UW3	Huang *et al*. [98]
Naphthalene	Barmultra grass (*Lolium multiflorum*)	*Pseudomonas putida* PCL1444	Kuiper *et al*. [85,86]
Phenanthracene	Barley (*Hordeum sativum* L.)	Degrading rhizosphere colonizing *Pseudomonas*	Ankohina *et al*. [99]
Chrysene	White Clover (*Trifolium repens* L.)	PAH tolerant *Rhizobium leguminosarum*	Johnson *et al*. [100]
